# Biochemical characterization of a new nicotinamidase from an unclassified bacterium thriving in a geothermal water stream microbial mat community

**DOI:** 10.1371/journal.pone.0181561

**Published:** 2017-07-27

**Authors:** Rubén Zapata-Pérez, Ana-Belén Martínez-Moñino, Antonio-Ginés García-Saura, Juana Cabanes, Hideto Takami, Álvaro Sánchez-Ferrer

**Affiliations:** 1 Department of Biochemistry and Molecular Biology-A, Faculty of Biology, Regional Campus of International Excellence “Campus Mare Nostrum”, University of Murcia, Murcia, Spain; 2 Murcia Biomedical Research Institute (IMIB), Murcia, Spain; 3 Microbial Genome Research Group, Yokohama Institute, JAMSTEC, Kanazawa, Yokohama, Japan; Universidad Autonoma de Madrid Centro de Biologia Molecular Severo Ochoa, SPAIN

## Abstract

Nicotinamidases are amidohydrolases that convert nicotinamide into nicotinic acid, contributing to NAD^+^ homeostasis in most organisms. In order to increase the number of nicotinamidases described to date, this manuscript characterizes a nicotinamidase obtained from a metagenomic library fosmid clone (JFF054_F02) obtained from a geothermal water stream microbial mat community in a Japanese epithermal mine. The enzyme showed an optimum temperature of 90°C, making it the first hyperthermophilic bacterial nicotinamidase to be characterized, since the phylogenetic analysis of this fosmid clone placed it in a clade of uncultured geothermal bacteria. The enzyme, named as UbNic, not only showed an alkaline optimum pH, but also a biphasic pH dependence of its *k*_*cat*_, with a maximum at pH 9.5–10.0. The two p*K*_a_ values obtained were 4.2 and 8.6 for p*K*_es1_ and p*K*_es2_, respectively. These results suggest a possible flexible catalytic mechanism for nicotinamidases, which reconciles the two previously proposed mechanisms. In addition, the enzyme showed a high catalytic efficiency, not only toward nicotinamide, but also toward other nicotinamide analogs. Its mutational analysis showed that a tryptophan (W83) is needed in one of the faces of the active site to maintain low *K*_*m*_ values toward all the substrates tested. Furthermore, UbNic proved to contain a Fe^2+^ ion in its metal binding site, and was revealed to belong to a new nicotinamidase subgroup. All these characteristics, together with its high pH- and thermal stability, distinguish UbNic from previously described nicotinamidases, and suggest that a wide diversity of enzymes remains to be discovered in extreme environments.

## Introduction

Nicotinamide adenine dinucleotide (NAD) is a cofactor required for numerous redox reactions, being its oxidized (NAD^+^)/reduced (NADH) ratio crucial for cell viability [1, 2]. The strategies that cells use to maintain this NAD^+^ homeostasis are quite elaborate [3] and differ between humans and most prokaryotes, but also between unicellular and multicellular eukaryotes. Nicotinamide (NAM) is the product of multiple NAD^+^-consuming enzymes, such as sirtuins, that are widely distributed in biology. In most organisms, NAM is converted to nicotinic acid (NA) by the enzyme nicotinamidase (EC 3.5.1.19) as the first step to recycling it into NAD^+^. However, human and mammalian genomes do not encode nicotinamidases and convert NAM directly into nicotinamide mononucleotide (NMN), which is then adenylated back to NAD^+^ in a step catalyzed by NMN adenylyltransferase (EC 2.7.7.1). This absence of nicotinamidase activity in humans and its crucial role in the NAD^+^ salvage pathways of not only the human pathogens involved in Lyme disease [4] and infantile visceral leishmaniasis [5], but also in an epizootic disease of domestic ducks caused by *Riemerella anatipestifer* [6], makes this enzyme a promising drug target.

In addition, there is a growing interest in using nicotinamidases in enzyme-coupled assays to identify modulators of sirtuins, which are relevant biomedical enzymes involved in lifespan, cancer, obesity and neurodegenerative diseases [7]. Nicotinamidases are also used in the biotechnological production of O-acetyl-ADP ribose (OAADPr) [8], a substrate of macrodomain enzymes, which have been the focus of recent interest for their biomedical implications [9]. OAADPr is produced by sirtuins through its deacetylation reaction using NAD^+^ and a lysine-acetylated peptide/protein as substrates, rendering NAM as a product. Since NAM inhibits the sirtuin deacetylation reaction, nicotinamidase is usually added to the mixture in order to avoid this phenomenon by fully converting NAD^+^ into OAADPr [8]. However, the above-described applications are now restricted to an academic context, since no inexpensive and stable source of enzyme has been found and no commercial nicotinamidases are available.

The first nicotinamidase activity was reported in 1952 [10], since when the activity of 24 more of these enzymes has been measured [6, 11–37]. However, their catalytic efficiency toward their respective natural substrate, nicotinamide, has only been reported in 12 of them [11, 12, 14, 16–18, 29, 31], and only in 6 of these also toward other nicotinamide analogs [11, 16, 17, 20, 29, 31]. Given that more than 8300 nicotinamidase sequences derived from large-scale sequencing and metagenomics have been deposited in the UniProt database, the number of nicotinamidases described to date represents only a 0.2% of the total. In addition, almost all nicotinamidases characterized come from mesophilic microorganisms, with the only exception of two extremophile archaeal nicotinamidases, one from *Pyrococcus horikoshii* (PhNic) [13] and the other from *Acidilobus saccharovorans* (AsNic) [16].

Taking into account the relevance of metagenomic approaches for identifying new enzymes, this work describes the characterization of a novel metagenomic nicotinamidase from an unclassified bacterium (UbNic), which was obtained by *in silico* identification of a nicotinamidase sequence in an already sequenced metagenomic library derived from a microbial mat formation taken from a subsurface geothermal water stream of the Hishikari epithermal mine in Japan [38]. This putative nicotinamidase/pyrazinamidase sequence was found in the fosmid clone JFF054_F02, and its functional screening showed high activity toward pyrazinamide as substrate. The heterologous expression and its kinetic study revealed that this enzyme is active over a broad range of pH values (from 5 to 11) and temperatures (up to 90°C), being the first bacterial nicotinamidase with such high optimum temperature and pH. In addition, UbNic is the second bacterial nicotinamidase described containing Fe^2+^ in its metal binding site, together with that of *Mycobacterium tuberculosis* [33], with which it also shares a similar metal binding domain sequence. Finally, its mutational analysis showed the important role of several amino acids in its catalytic efficiency toward different substrates.

## Materials and methods

### Cloning and mutagenesis of the UbNic gene

Genomic DNA was obtained from an environmental sample acquired in a geothermal water stream microbial mat community located at the deepest level (320 meters) of the Japanese epithermal mine [38]. We used the fosmid library prepared in the previous study by the following protocol [38]. High molecular weight genomic DNA extracted from the discharge point was loaded on pulse-field agarose gel electrophoresis after both DNA ends were repaired by End-It DNA End-Repair Kit (Epicentre, Madison; USA). After electrophoresis was complete, an agarose plug containing 33–48 kb DNA was cut out. Genomic DNA purified from this plug was cloned into pCC1FOS (Epicentre). The ligated fosmids (5280 in total) were packaged into MaxPlax Lambda Packaging Extract (Epicentre) and the packaged particles were transferred into *Escherichia coli* EPI 300 (Epicentre), with an average insert size of 37 kb.

We used genetic information of the fosmid library obtained in the previous study by the following strategies [39]. Fosmid clones were sequenced by the Sanger method using ABI PRISM 3730 and MegaBase100 (GE Healthcare, Japan) DNA sequencers. Sanger sequence reads were assembled using Phrap with default parameters. Final gaps in the clone were closed by direct sequencing of the PCR products. CDS were predicted using a combination of the Genome Gambler^™^ and MetaGeneAnnotator programs [40, 41]. The amino acid sequences of predicted CDSs were subjected to a BLASTP homology search against the protein databases for functional assignment. Significant homology was defined as at least 30% identity over 60% of the CDS; however, those CDSs showing <30% identity over >60% of the protein were also included, as previously described [42].

After sequencing, a putative nicotinamidase sequence was found in fosmid JFF054_F02. Then, the clone containing the latter fosmid was checked for nicotinamidase activity using the previously described pyrazinamide/ammonium ferrous sulfate method [11]. Briefly, after growing fosmids at 37°C overnight, their pellets were resuspended in a mixture of 20 mM pyrazinamide and 1% ammonium ferrous sulfate dissolved in MilliQ water, and incubated for 1 h at 37°C until an intense orange-red color appeared. Finally, the fosmid was used as the source for the uncultured bacterium nicotinamidase gene (Uniprot code: H5SPS2). The gene was amplified by polymerase chain reaction (PCR) and engineered to contain *Nhe*I and *Xho*I restriction sites using the primers listed in S1 Table. The resulting PCR product was purified and digested with the corresponding restriction enzymes and cloned into pET28a vector (Novagen), which carries an N-terminal 6-histidine tag. After sequencing, a selected clone harboring the correct sequence of the gene was denoted as pET28a-UbNic.

Four point mutations (H75E, H75S, W83F and A151C) were introduced by site-directed mutagenesis using overlap extension PCR [43]. The primers used for mutagenesis are also listed in S1 Table. PCR products were digested with *Dpn*I (New England Biolabs) to ensure complete removal of the methylated parental DNA prior to transformation. All mutations were confirmed by sequencing.

### Enzyme overexpression and purification

UbNic wild type and four mutants were produced in *Escherichia coli* Rosetta 2 (DE3)pLysS carrying the recombinant plasmid pET28a-UbNic. Cells were cultured in 1 liter of Terrific Broth (TB) supplemented with kanamycin (50 μg/mL) and chloramphenicol (34 μg/mL) at 37°C. When OD_600_ reached a value of 4, the culture was induced with 0.1 mM isopropyl-β-thiogalactoside (IPTG) during 16 hours at 25°C. The induced culture was harvested by centrifugation. Cells were resuspended in lysis buffer (50 mM phosphate buffer pH 7.3 containing 300 mM NaCl and 1 mM phenylmethylsulfonyl fluoride) and disrupted using a Bead Beater (BioSpec, USA). The protein in the supernatant was then purified by Ni^2+^-chelating affinity chromatography (ÄKTA Prime Plus, GE Lifesciences) onto a HiPrep IMAC 16/10 FF 20 mL column (GE Lifesciences), eluted with imidazol and gel filtrated onto a Superdex 200 HiLoad 16/600 column (GE Lifesciences), thus obtaining an electrophoretically pure enzyme. The protein molecular mass was determined by SDS-PAGE, analytical gel filtration and HPLC/ESI-MS [44]. Protein concentration was determined using Bradford reagent (Bio-Rad) and BSA as standard.

### Enzyme assays

Nicotinamidase activity was measured by HPLC (Agilent 1100 series) using a reverse-phase C18 250x4.6 mm column (Gemini C18, Phenomenex) and a mobile phase consisting in 20 mM ammonium acetate pH 6.9 running at 1 mL/min. The standard reaction medium contained 1 mM NAM and 40 nM of purified UbNic in 100 mM sodium phosphate buffer pH 7.3. Reactions were stopped by the addition of trifluoroacetic acid at 1% (v/v) concentration. Under these conditions, the retention time (R_T_) for NAM and NA were 19.9 and 7 minutes, respectively. One unit of activity was defined as the amount of enzyme required to cleave 1 μmol of NAM releasing 1 μmol of nicotinic acid in 1 min. The activity toward other NAM analogs was also determined by HPLC. Finally, the inhibition constants toward nicotinaldehyde and 5- bromo-nicotinaldehyde were obtained as previously described [18], using Morrison´s quadratic equation [45], which is formulated for for tight-binding inhibitors with intrinsic Ki values < 5 μM.

The effect of pH and pH-stability toward NAM was determined at 37°C in 100 mM sodium acetate (pH 4.0–5.0), sodium phosphate (pH 6.0–7.3), Tris-HCl (pH 8.0), glycine (pH 9.0–10.0) and CAPS (pH 11) or in 100 mM Citric-Hepes-CHES continuous buffer. In addition, the kinetic parameters were measured in the pH range 4.0–10.0 using buffers of constant ionic strength [29, 46]. In this case, TBA buffer (50 mM Tris, 50 mM BisTris, 100 mM sodium acetate) was used in the pH range 4.0–8.5, and ATE buffer (100 mM ACES, 52 mM Tris, 52 mM ethanolamine) was used in the pH range 8.0–10.0. The experimental data of *k*_*cat*_ versus pH was fitted to the following equation [47, 48]:

$$\text{log y}=\text{log}\left[\frac{V\;\left(1+\frac{aK_{es2}}{H}\right)}{1+\left(\frac{H}{K_{es1}}\right)+\left(\frac{K_{es2}}{H}\right)}\right]$$

using GraphPad Prism to determine p*K*_a_ values, where H is the proton concentration, *K* represents the protonation equilibria for the enzyme-substrate complex, *a* is the pH-independent value of *y*, and V is the kinetic parameter (*k*_*cat*_).

### Thermal shift assay

This assay is a fluorescence-based technique that measures the denaturation or melting temperature (*T*_*m*_) of protein samples. In the presence of a small molecule, the thermal shift assay (TSA) relies on the fact that a ligand binding may stabilize proteins to thermal denaturation [49]. Protein melting curves were determined using Sypro Orange fluorescent dye (Molecular Probes), as previously described [44] in a 7500 Real-Time PCR equipment (Applied Biosystems), increasing the temperature from 25 to 95°C in 1°C steps every 60 seconds in the presence of 40X of the fluorescent dye (emission at 530 nm and excitation at 490 nm) and a final protein concentration of 1 μM.

### Determination of metal ion content

Enzyme metal content was determined by inductively coupled plasma-optical emission spectrometry (ICP-OES) using Optima 2000 DV equipment (Perkin-Elmer, MA, USA). Five mL of purified UbNic were diluted to a final concentration of 48 μM in five mL of HNO_3_ (69%) and incubated for 4 hours at 85°C, as previously described [33]. Samples were analyzed in triplicate runs. The metal ion content of the protein was calculated using the calibration curve obtained for each metal ion (Fe^2+^, Zn^2+^ and Mn^2+^) after subtracting the background signal in the blank-buffer HNO_3_ mixture.

### *In silico* analysis

BLAST searches were used to identify homologs of nicotinamidases [50]. Sequences were aligned and displayed using MAFFT [51] and ESPript [52], respectively. Protein sequences were 3D modelled with Swiss-Model [53]. Phylogenetic tree was obtained using MEGA 7.0 [54] and displayed with iTOL [55].

## Results and discussion

### Amino acid sequence comparison and phylogenetic analysis

The metagenomic library obtained from a water stream located at the deepest level of the Japanese epithermal mine (320 meters below the land surface), when sequenced [38], revealed the presence of an uncultured bacterium nicotinamidase (UbNic) in the fosmid clone JFF054_F02 (Accession: AP011794). Later hierarchical clustering of its 16S rRNA placed JFF054_F02 correctly in a clade corresponding to unclassified bacteria similar to GAL08 [39]. This clade groups unclassified bacteria from geothermal systems, such as those found in Yellowstone National Park (GAL08) and in the hot spring 2 in the Rupite basin (Bulgaria; isolate S2R-72) [39]. However and unfortunately, JFF054_F02 was incorrectly annotated in the databases as coming from an uncultured *Acidobacteria bacterium* (Uniprot code: H5SPS2 and GenBank: BAL58158.1), this error being persisting in the databases. In addition and more interestingly, Takami *et al*. [39] showed that the uncultured bacteria corresponding to this GAL08 clade have a high estimated maximum temperature for growth (~86°C) based on the correlation between the maximum growth temperature and the G+C contents of its 16S rRNA gene, indicating the possibility of finding extremozymes in the fosmid clone JFF054_F02.

When the amino acid sequence of UbNic was first compared with other characterized nicotinamidases/pyrazinamidases, its sequence alignment indicated that UbNic has high sequence identity with isochorismatase hydrolases, a subfamily within the cysteine-hydrolases superfamily. UbNic showed 53% identity with the archaeon *Pyrococcus horikoshii* nicotinamidase (PhNic, PDB codes: 1ILW, 1IM5) [13] and was found to have 42% and 40% identity with the nicotinamidases from the gamma-proteobacteria *Acinetobacter baumanii* (AbNic, PDB codes: 2WT9, 2WTA) [20] and the actinobacteria *Mycobacterium tuberculosis* (MtNic, PDB code: 3PL1) [33], respectively. Surprisingly, UbNic also showed a relevant degree of identity (38%) with the eukaryotic nicotinamidase from *Leishmania infantum* (LiNic, PDB code: 3R2J) [5], which was higher than those found for *Acidilobus saccharovorans* (AsNic, 33%) [16] and *Oceanobacillus iheyensis* (OiNic, 28%) nicotinamidases [17].

**Fig 1:**
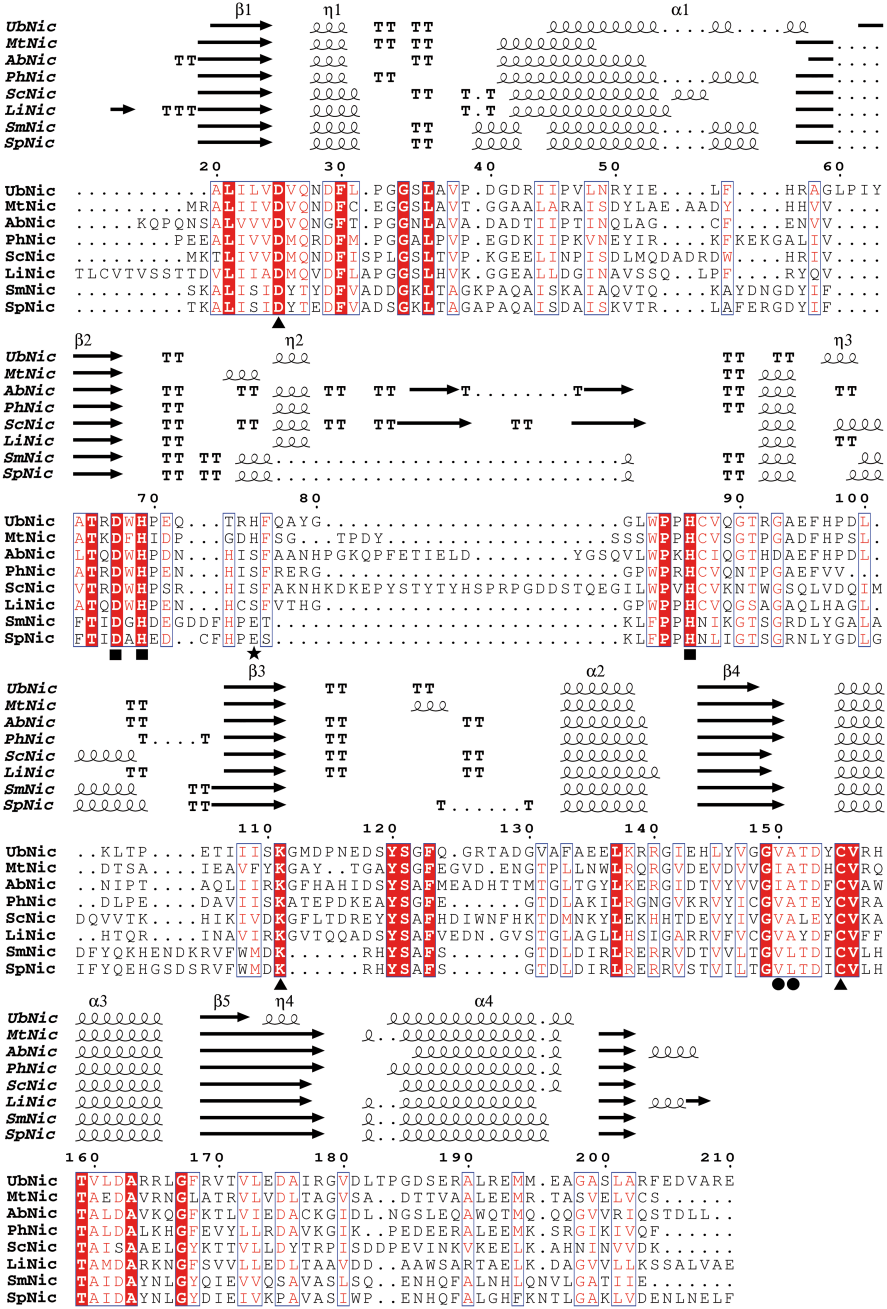
Multiple sequence alignment between UbNic and related nicotinamidases. Symbols above blocks of sequences represent the secondary structure. Springs, arrows and TT represent helices, strands and strict β-turns, respectively. Strictly conserved amino acids across nicotinamidases have a red background and similar residues are marked with a rectangle. Residues involved in catalysis (▲), in *cis*-peptide bond (●) and in the metal ion binding (■) are also shown. The fourth amino acid involved in metal binding is shown as a star (★). Crystallized nicotinamidases (Nic) were denoted as MtNic for *Mycobacterium tuberculosis* Nic (PDB: 3PL1), AbNic for *Acinetobacter baumanii* Nic (PDB: 2WT9), PhNic for *Pyrococcus horikoshii* Nic (PDB: 1IM5), ScNic for *Saccharomyces cerevisiae* Nic (PDB: 2H0R), LiNic for *Leishmania infantum* Nic (PDB: 3R2J), SmNic for *Streptococcus mutans* Nic (PDB: 3S2S) and SpNic for *Streptococcus pneumoniae* Nic (PDB: 3O90).

Sequence alignment also revealed that UbNic includes the totally conserved residues that form the catalytic triad of the cysteine hydrolases family (Fig 1, triangles), the catalytic cysteine (C155), aspartate (D25) and lysine (K111) [13, 18, 24, 32]. UbNic also presents the characteristic amino acids that form the *cis*-peptide bond found in the active center of all described nicotinamidases, which in the case of UbNic are V150 and A151 (Fig 1, circles), preceded by a conserved glycine (G149). In addition, UbNic also has the typical four-amino acid nicotinamidase metal ion-binding motif, which is invariably composed by one aspartate (D67) and two histidines (H69 and H86) (Fig 1, squares). However, the fourth amino acid depends on the metal ion that is bound and on the structure of the protein, and it is usually a glutamic acid, a serine or a histidine [13, 32, 33]. In the case of UbNic, its structural alignment with other crystallized nicotinamidases showed that this fourth residue is a histidine (H75) (Fig 1, star), being similar to the histidine described in the pyrazinamidases from *Mycobacterium tuberculosis* [33] and *Mycobacterium smegmatis* (MsNic) [19]. Finally, other residues forming the active site hydrophobic cavity were also found in UbNic, and correspond with residues F30, L36, W83, G112, Y120 and Y154.

**Fig 2:**
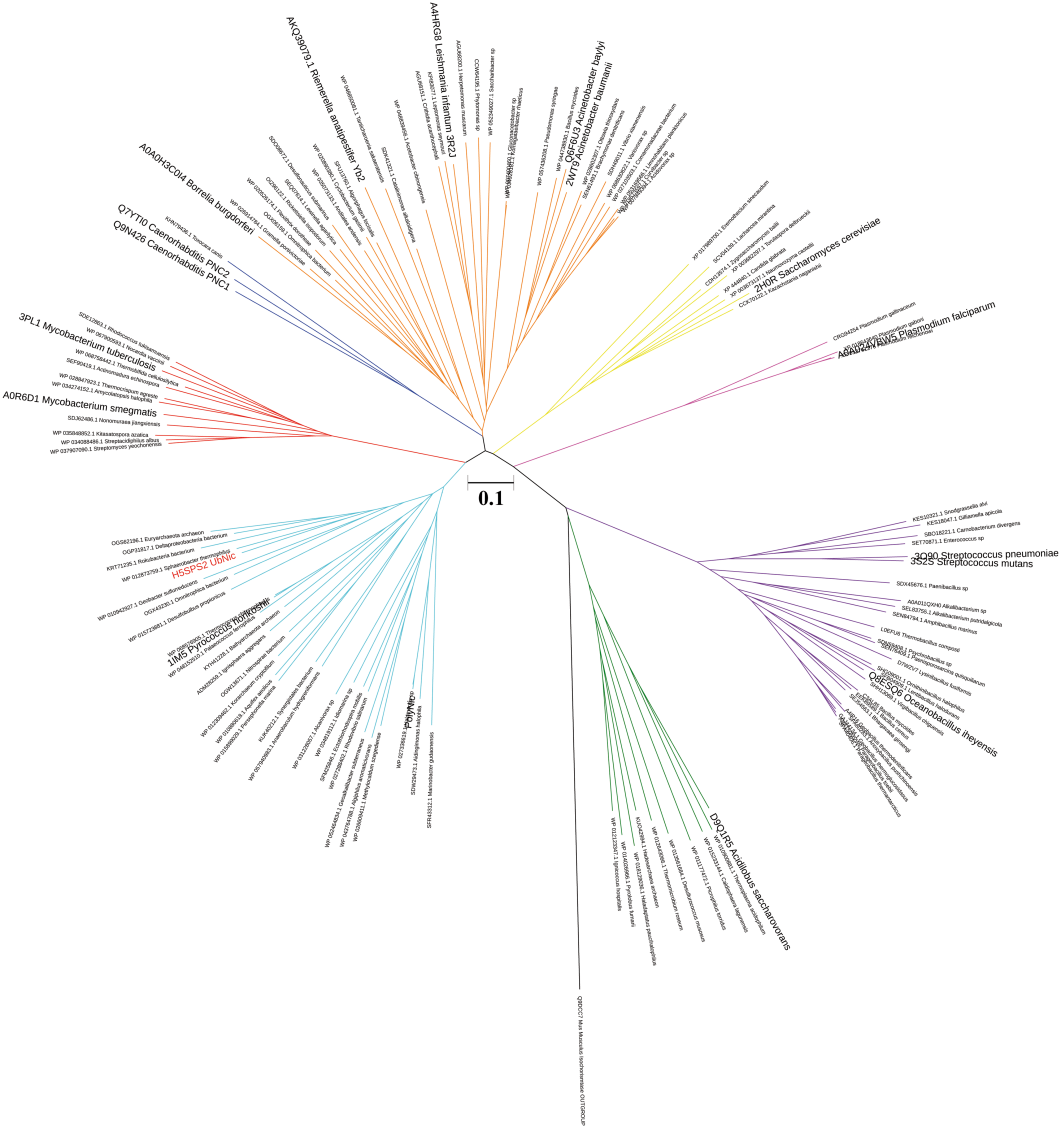
Unrooted phylogenetic tree of UbNic and other related nicotinamidases illustrating evolutionary relationships among them. *Mus musculus* isochorismatase was used as outgroup. Alignments were carried out with CLUSTALW algorithm and the phylogenetic tree was constructed using a neighbor-joining analysis in MEGA 7.0 [54]. Different groups are marked in different colors. Already described nicotinamidases are in magnified letters and those crystallized shows their PDB code. The sequences used are listed in S2 Table.

The phylogenetic analysis carried out with MEGA [54] showed that UbNic clusters in the same clade as the archaeal *Pyrococcus horikoshii* nicotinamidase [13] and the recently described polygenomic nicotinamidase PolyNic [11] (Fig 2, cyan). This clade contains a variety of sediment-growing and thermophilic microorganisms from both the Bacteria and the Archaea domains. Interestingly, UbNic, PolyNic and PhNic are situated in a different clade from that formed by thermophilic archaeal nicotinamidases, which includes that from *Acidilobus saccharovorans* [16] (Fig 2, green). Other already described nicotinamidases are distributed in different clades throughout the phylogenetic tree, including a clade for yeasts (Fig 2, yellow) and nematodes (Fig 2, blue), where *Saccharomyces cerevisiae* and *Caenorhabditis elegans* nicotinamidases are included [18, 24, 26, 28, 29, 56], respectively. The nicotinamidases from pathogenic microorganisms are distributed in three different clades, one including those of *Borrelia burgdorferi*, *Leishmania infantum*, *Acinetobacter baumanii* and the recently described nicotinamidase from *Riemerella anatipestifer* [4–6, 18, 20] (Fig 2, orange); and the others containing those of *Mycobacterium tuberculosis* [31–33] (Fig 2, red), and *Streptococcus pneumoniae* [18, 34] (Fig 2, purple), respectively. Finally, it is worth mentioning that the clade described by *Plasmodium falciparum* nicotinamidase [18] and related members of the *Plasmodium* genus represented an special case in the phylogenetic tree (Fig 2, pink), since their sequences are longer than those of the rest of nicotinamidases, showing an N-ter extension of about 200 amino acids of unknown function.

### Biochemical characterization

After sequence-based metagenomics and phylogenetic analysis, JFF054_F02 fosmid was tested with the whole-cell functional screening method recently described for identification of new nicotinamidases from fosmid metagenomic/polygenomic libraries method [11]. As shown in S1 Fig, when the clone was induced with autoinduction solution (Epicentre, USA) and revealed with the pyrazinamide/ammonium ferrous sulfate method [11], an intense orange-red color was detected, indicating not only the presence of pyrazinamidase activity, but also good expression in *E*. *coli*. This color was also more intense than that of PolyNic under the same conditions (S1 Fig). The same higher expression was observed when the gene encoding the nicotinamidase from this unclassified bacterium was subcloned into pET28a vector, as described in Materials and Methods, and the resultant protein purified to homogeneity with a yield of 75 mg of UbNic per liter of culture. The molecular mass of the purified protein was determined by gel filtration (21.2 kDa), by HPLC/ESI-MS (23.9 kDa) and by bioinformatic tools (26.4 kDa) [57], confirming the monomeric nature of the enzyme.

**Fig 3:**
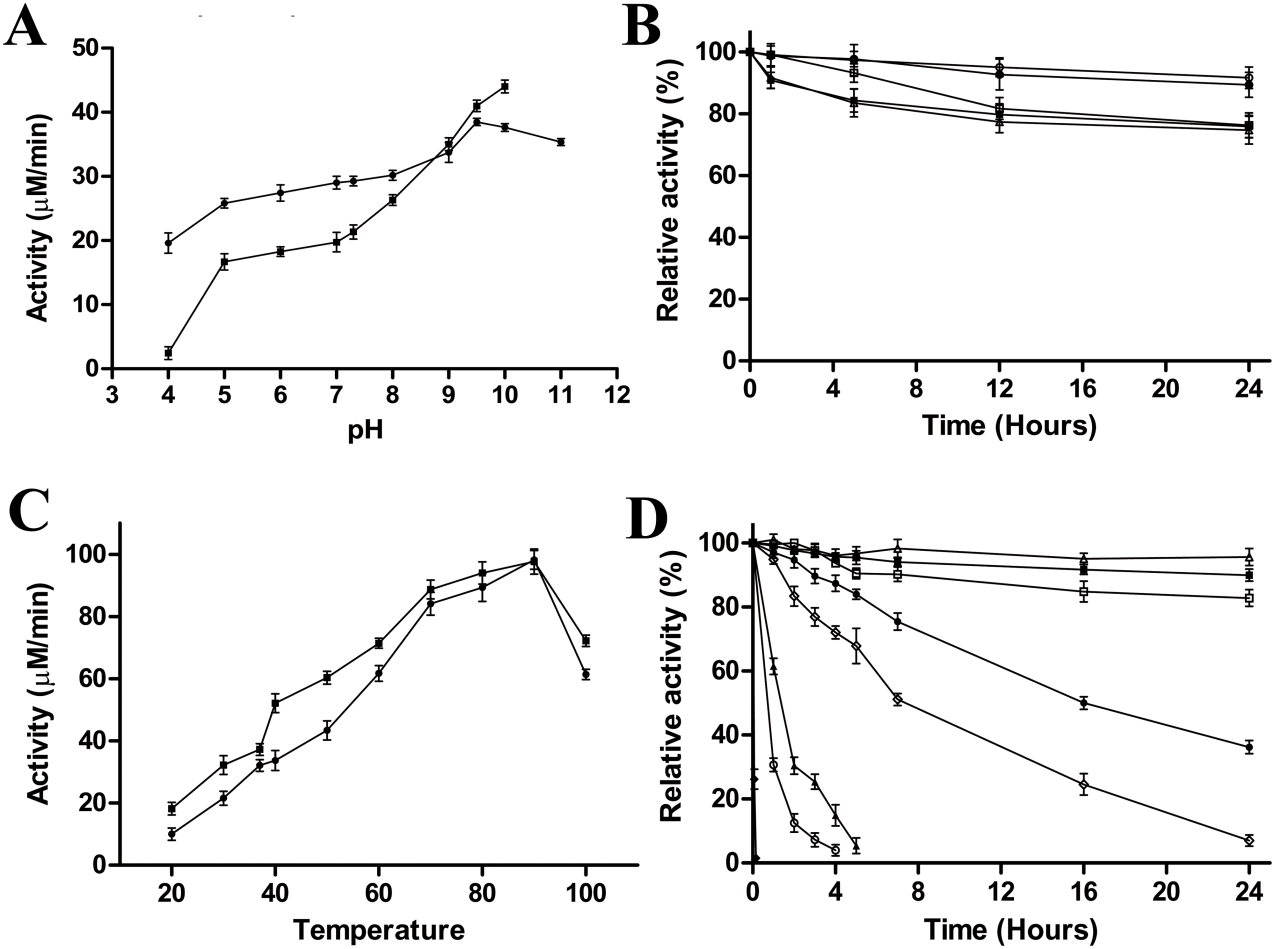
Effect of pH and temperature on UbNic activity and stability. A) pH profile for UbNic determined by HPLC in the following 100 mM discrete buffers (sodium acetate pH 4.0–5.0, sodium phosphate pH 6.0–7.3, Tris-HCl pH 8.0, glycine pH 9.0–10.0 and CAPS pH 11.0) (●) or in 100 mM Citric-Hepes-CHES continuous buffer (■). The assay conditions at 37°C were 1 mM nicotinamide and 40 nM of UbNic. B) pH stability. UbNic was incubated at 37°C in different 100 mM buffers: sodium acetate pH 5.0 (Δ) and pH 6.0 (■), sodium phosphate pH 7.3 (○), Tris-HCl pH 8.0 (□) and glycine pH 9.5 (●). Residual activity was measured by HPLC under the standard reaction conditions. C) Temperature profile in 100 mM sodium phosphate pH 7.3 (●) or 100 mM glycine pH 9.5 (■). Activity was measured as described above from 20 to 100°C. D) Temperature stability. UbNic was incubated at 4°C (Δ), 20°C (■), 37°C (□), 50°C (●), 60°C (◊), 70°C (▲), 80°C (○) and 90°C (♦) in 100 mM sodium phosphate buffer pH 7.3. Residual activity was measured by HPLC under the standard reaction conditions. Data points are the average of three repeated experiments.

The activity of the recombinant enzyme was pH-dependent with a maximum at pH 9.5 in glycine buffer or at pH 10.0 when using Citric-HEPES-CHES buffer (Fig 3A). However, in this continuous buffer, the activity was lower at all the pHs tested compared with discrete buffers (Fig 3A, squares), except at pH 9.5 and 10.0. In fact, from pH 5.0–7.3, the enzyme showed 1.5-fold less activity in the Citric-HEPES-CHES buffer (Fig 3A, squares) than in the discrete buffers (Fig 3A, circles). This difference in activity was more pronounced at pH 4.0, at which the enzyme was almost inactive in the continuous buffer (Fig 3A, squares). This alkaliphilic optimum pH (9.5–10.0) is a noteworthy characteristic of UbNic, since most of described nicotinamidases in the bibliography have a neutral optimum pH (6.0–7.5) [14, 16–18, 22, 23]. The only exception to this usual neutral optimum pH in nicotinamidases is the recently described nicotinamidase found in a polygenomic library (PolyNic), which also has an optimum pH of 10.0 [11]. However, PolyNic has a sharper pH profile compared with UbNic, showing no activity at pH 4.0 and a drastic decrease in activity at pH values higher than pH 10.0 [11].

The stability of UbNic at different pHs was studied at 37°C for comparative purposes. The results showed that UbNic was a very stable nicotinamidase from acid (pH 5.0) to basic pHs (up to pH 9.5) (Fig 3B). Its stability was higher in sodium phosphate pH 7.3 (Fig 3B, open circles) and in glycine pH 9.5 (Fig 3B, filled circles), maintaining 90% activity after 24 hours of incubation at such pHs. At lower pH values, UbNic was found to be less stable, although it maintained 75% activity in sodium acetate pH 5.0 (Fig 3B, open triangles) and pH 6.0 (Fig 3B, filled squares) after 24 hours. This stability at acidic pHs could be explained by the weakly acidic conditions (pH 5.1) found in the hot water stream overlaying the microbial mat community containing this unclassified bacterium [58]. Surprisingly, the enzyme was less stable than expected in Tris-HCl pH 8.0 (Fig 3B, open squares), showing a behavior similar to that found at pH 5.0 and 6.0. When its pH-stability was compared with the only data available for OiNic at 37°C [17], UbNic was more stable at all the pHs studied, since OiNic lost 50% of its activity in only 9.5 hours at pH 7.3 [17], being this decrease in activity even more drastic at other pHs in comparison with UbNic.

The temperature also affected UbNic activity, and surprisingly, the enzyme showed its maximal activity at 90°C, both at pH 7.3 (Fig 3C, circles) and pH 9.5 (Fig 3C, squares). This high optimum temperature has only been found in the nicotinamidase from the thermophilic archaeon *Acidilobus sacchavorans* (AsNic) [16]. The rest of nicotinamidases have optimum temperatures ranging from 25 to 50°C [17, 18, 22], including PolyNic, which has its optimum temperature at 50°C, with a sharp decrease in activity at 60°C [11]. However, UbNic stability at 90°C and pH 7.3 was very low, since it completely lost the activity in just 10 minutes (Fig 3D, diamonds). By contrast, when the temperature decreased from 80°C to 50°C (Fig 3D), its half-life increased from 0.7 to 17 hours. In fact, at 60°C, the half-life of UbNic is 2.3-fold higher than that found for the thermophilic AsNic (7 *vs* 3 h) [16]. Lower incubation temperatures increased UbNic stability even more, with only a 17% and 3% decrease in activity after 24 hours at 37°C and 4°C (Fig 3D), respectively. Indeed, its half-life at 37°C was 4.5 days, which is 14.5-fold higher than that described for OiNic [17]. This stability was more pronounced at 4°C, since UbNic retained a 64% of its activity after 43 days.

**Fig 4:**
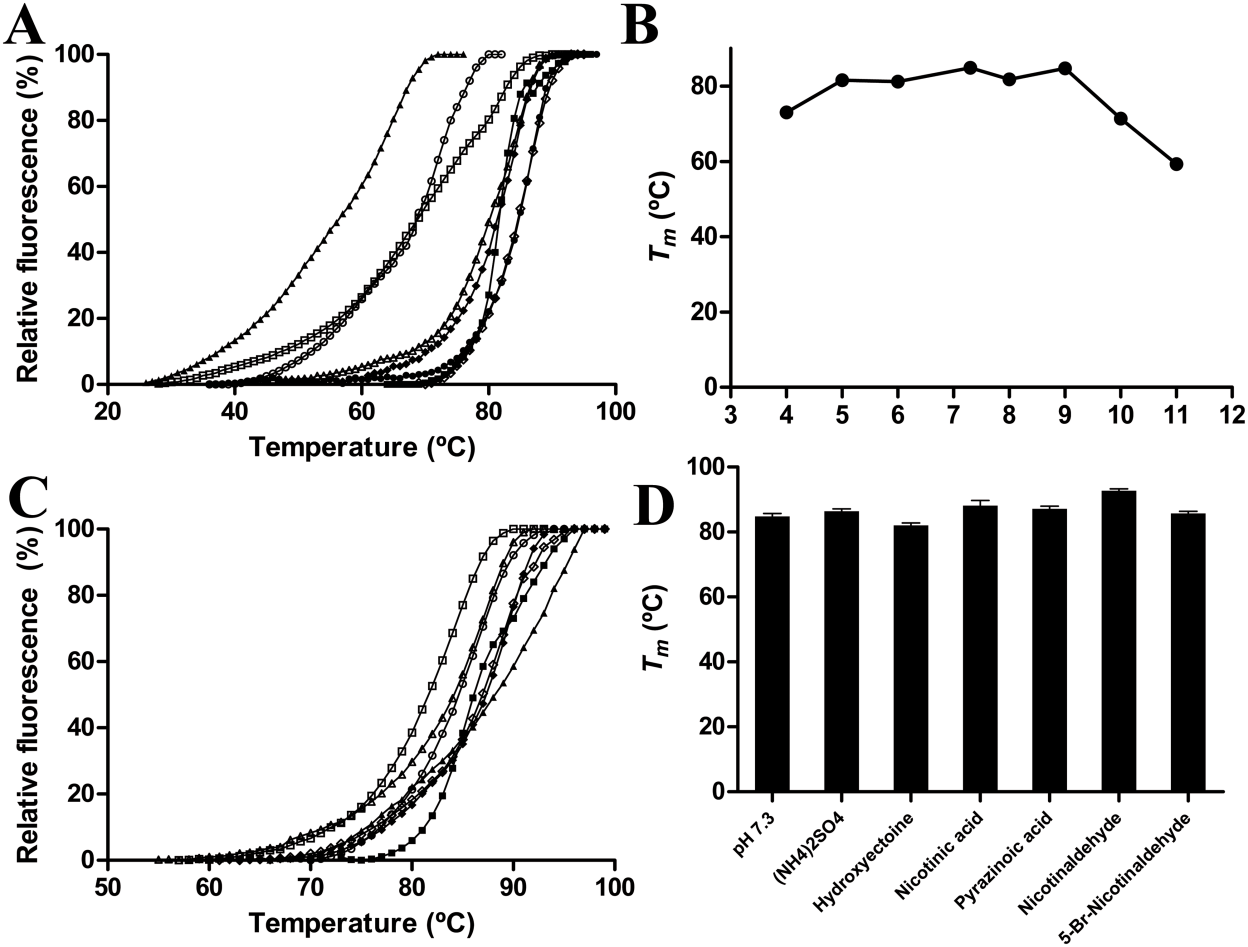
Thermal shift assay of UbNic. A) Melting temperature curves obtained in different buffers. The buffers used (100 mM) were sodium acetate pH 4.0 (○) and 5.0 (■), sodium phosphate pH 6.0 (Δ) and 7.3 (◊), Tris-HCl pH 8.0 (♦), glycine pH 9.0 (●) and 10.0 (□), and CAPS pH 11.0 (▲). B) UbNic melting temperature profile at different pHs. C) Effect of different modulators on UbNic melting temperature. Melting curves were obtained in presence of 100 mM sodium phosphate pH 7.3 (○); protein stabilizers, such as ammonium sulfate (■) and hydroxyectoine (□); nicotinamidase reaction products, such as nicotinic (♦) and pyrazinoic (◊) acids; and nicotinamidase inhibitors, such as nicotinaldehyde (▲) and 5-bromo-nicotinaldehyde (Δ). D) UbNic melting temperatures obtained with the different modulators described in C. Data points are the average of three repeated experiments.

The above-described high stability shown by UbNic was also corroborated by a thermal shift assay (TSA) (Fig 4), in which melting temperatures (*T*_*m*_) above 84°C were found for the enzyme in sodium phosphate buffer pH 7.3 (*T*_*m*_ = 84.8±0.3°C) and in glycine buffer pH 9.0 (*T*_*m*_ = 84.7±0.2°C) (Fig 4A and 4B). At extreme high or low pH values, the enzyme was less stable, especially in CAPS pH 11.0 (*T*_*m*_ = 59.3±0.2°C), glycine pH 10.0 (*T*_*m*_ = 71.3±0.1°C) and sodium acetate pH 4.0 (*T*_*m*_ = 73.1±0.3°C) (Fig 4B). The enzyme also showed a lower melting temperature than expected at pH 8.0 (*T*_*m*_ = 81.8±0.2°C), probably because Tris-HCl is not as good as other buffers (phosphate or glycine) for UbNic stability. Collectively, the results obtained in the thermal shift assay correlate well with the pH stability curves obtained in Fig 3B. In addition, the effect of different compounds on the *T*_*m*_ was also studied (Fig 4C and 4D). As regards protein stabilizers, only ammonium sulfate at 1 M was able to produce a slight increase in its *T*_*m*_ (*T*_*m*_ = 86.4±0.3°C) in comparison with that found in sodium phosphate pH 7.3, whereas hydroxyectoine (*T*_*m*_ = 82.1±0.4°C) did not (Fig 4C). The nicotinamidase reaction products, nicotinic and pyrazinoic acids, also improved UbNic stability, increasing its *T*_*m*_ to 87.7±0.1°C and 87.1±0.2°C (Fig 4C), respectively. These results indicate their binding to UbNic, as it has been described for other proteins [49]. In the same way, the nicotinamidase inhibitors, nicotinaldehyde and 5-bromo-nicotinaldehyde, also increased *T*_*m*_ up to 92.7±0.3°C and 85.7±0.1°C (Fig 4D), respectively. Finally, when the above-mentioned melting temperatures were compared with other nicotinamidases, UbNic showed the highest *T*_*m*_ described for a nicotinamidase, its value being 31°C and 41°C higher than those from *Oceanobacillus iheyensis* and *Mycobacterium tuberculosis* [17, 33], respectively. In addition, the increase in melting temperature (7°C) obtained with nicotinaldehyde (Fig 4D) was similar to that found for OiNic [17].

### Substrate specificity and kinetic parameters

UbNic substrate specificity was assayed at 37°C with different compounds, including nicotinamide and its analogs pyrazinamide, 5-methyl-nicotinamide and two nicotinate esters (methylnicotinate and ethylnicotinate) (Table 1). UbNic showed a clear preference for nicotinamide (*k*_*cat*_*/K*_*m*_ = 189.2 mM^-1^·s^-1^) compared with the rest of the analogs. In fact, the enzyme expressed a 3.3-fold higher catalytic efficiency for NAM than for the best analog compound (pyrazinamide) and a 67-fold higher than that found for the worst substrate (ethylnicotinate) (Table 1). However, and surprisingly, this catalytic efficiency for ethylnicotinate (*k*_*cat*_*/K*_*m*_ = 2.8 mM^-1^·s^-1^) was 2.8- and 28-fold higher than that described for PolyNic and OiNic [11, 17], respectively. To further study UbNic substrate specificity, four mutants were produced. The first was designed to modify one of the faces of the active site (W83F), the second to change the oxyanion hole at the active site (A151C) and the other two to alter the fourth and differential amino acid in metal binding (H75S and H75E). These latter mutations were conceived to cover all the known residues described for such positions in nicotinamidases.

**Table 1:** Kinetic parameters of UbNic wild type and its mutants.

	*K*_m_ (mM)	*k*_cat_ (s^-1^)	*k*_cat_/*K*_m_ (mM^-1^·s^-1^)
**Wild Type**			
NAM	0.12±0.02	22.7±0.4	189.2
PZA	0.33±0.05	18.7±0.3	56.7
5-methyl-NAM	1.09±0.20	43.7±0.3	40.1
Methylnicotinate	1.08±0.11	54.5±0.8	50.5
Ethylnicotinate	1.31±0.03	3.7±0.3	2.8
**H75S**			
NAM	0.23±0.03	12.5±0.1	54.3
PZA	0.59±0.07	1.3±0.1	2.2
5-methyl-NAM	0.23±0.03	12.4±0.3	53.9
Methylnicotinate	0.68±0.06	1.9±0.2	2.8
Ethylnicotinate	1.21±0.06	0.2±0.03	0.2
**H75E**			
NAM	0.15±0.01	10.2±0.4	68.0
PZA	0.67±0.18	3.5±0.2	5.2
5-methyl-NAM	0.14±0.01	4.9±0.4	35.0
Methylnicotinate	0.41±0.04	5.8±0.3	14.1
Ethylnicotinate	0.46±0.03	0.1±0.01	0.2
**W83F**			
NAM	0.39±0.04	15.5±0.1	39.7
PZA	2.89±0.82	3.1±0.4	1.1
5-methyl-NAM	0.74±0.08	13.7±0.3	18.5
Methylnicotinate	3.59±0.33	4.9±0.4	1.4
Ethylnicotinate	3.84±0.53	0.2±0.03	0.1
**A151C**			
NAM	0.10±0.01	5.1±0.2	51.0
PZA	0.22±0.01	7.5±0.4	34.1
5-methyl-NAM	0.21±0.05	9.6±0.4	45.7
Methylnicotinate	0.21±0.07	4.3±0.1	20.5
Ethylnicotinate	0.89±0.07	0.3±0.01	0.3

The mutants obtained showed a reduced catalytic efficiency for nicotinamide, which ranged from 2.8-fold for H75E to 4.8-fold for W83F. This reduction in catalytic efficiency was higher in the case of pyrazinamide, which had a greater effect on the *k*_*cat*_ in all the mutants, except A151C, where the *k*_*cat*_ was only reduced 2.5-fold compared with the 14.4-fold decrease in H75S. However, this latter mutant (H75S) showed an increase in the catalytic efficiency for 5-methyl-nicotinamide (*k*_*cat*_*/K*_*m*_ = 53.9 mM^-1^·s^-1^) compared with UbNic wild type (UbNic_WT_) (*k*_*cat*_*/K*_*m*_ = 40.1 mM^-1^·s^-1^), which was produced by a balanced reduction in both *K*_*m*_ and *k*_*cat*_ (Table 1). As regards nicotinate esters, A151C mutant was the best preserver of activity for both esters, whereas W83F showed a 36- to 28-fold decrease in activity compared with UbNic_WT_ for methylnicotinate and ethylnicotinate (Table 1), respectively. This was mainly due to a huge increase in the *K*_*m*_, probably due to the different size and hydrophobicity of phenylalanine compared with tryptophan. Interestingly, all mutants drastically reduced the *k*_*cat*_ toward ethylnicotinate, showing that any change in the active site perturbs the activity toward this poor substrate, hindering C155 thiolate nucleophilic attack at the C7 position of ethylnicotinate. Collectively, the results described for UbNic in Table 1 showed that, among the two mutations in the fourth amino acid involved in metal binding, H75E was better tolerated than H75S; that the change of alanine 151 for a cysteine did not excessively perturb the oxyanion hole; and that a tryptophan (W83) was needed on one of the faces of the active site to keep *K*_*m*_ values low with all the substrates tested.

When the catalytic efficiencies of UbNic were compared with those of other nicotinamidases, the *k*_*cat*_*/K*_*m*_ toward nicotinamide (*k*_*cat*_*/K*_*m*_ = 189.2 mM^-1^·s^-1^) at 37°C was one of the highest described in the bibliography for a non-pathogenic microorganism, being comparable to that from *Acinetobacter baylyi* at 30°C in Hepes buffer (*k*_*cat*_*/K*_*m*_ = 280 mM^-1^·s^-1^) [12]. It was also higher than those described for *Plasmodium falciparum* (*k*_*cat*_*/K*_*m*_ = 96 mM^-1^·s^-1^), *Borrelia burgdorferi* (*k*_*cat*_*/K*_*m*_ = 0.082 mM^-1^·s^-1^), *C*. *elegans* PNC1 (*k*_*cat*_*/K*_*m*_ = 27 mM^-1^·s^-1^) and *C*. *elegans* PNC2 (*k*_*cat*_*/K*_*m*_ = 3.6 mM^-1^·s^-1^) nicotinamidases [18]. However, it was lower than those of *Saccharomyces cerevisiae* (*k*_*cat*_*/K*_*m*_ = 358 mM^-1^·s^-1^) [18], *Streptococcus pneumoniae* (*k*_*cat*_*/K*_*m*_ = 777 mM^-1^·s^-1^) [18] and *Acidilobus saccharovorans* (*k*_*cat*_*/K*_*m*_ = 427 mM^-1^·s^-1^) [16] nicotinamidases, but in this last case, the kinetic parameters of this archaeal nicotinamidase were determined at 60°C. When UbNic activity was measured at this temperature, the catalytic efficiency of the enzyme increased 55-fold (*k*_*cat*_*/K*_*m*_ = 10425 mM^-1^·s^-1^) for nicotinamide compared to that observed at 37°C (S2 Fig). This increment in catalytic efficiency for nicotinamide was basically due to a 30-fold decrease in *K*_*m*_ (*K*_*m*_ = 4.0 ± 0.1 μM) with a slight increase in *k*_*cat*_ (1.8-fold; *k*_*cat*_ = 41.7 s^-1^). In light of this increase in the catalytic efficiency at 60°C for nicotinamide, its effect toward pyrazinamide was also studied (S2 Fig). The catalytic efficiency for this latter substrate also increased 19.5-fold (*k*_*cat*_*/K*_*m*_ = 1105.4 mM^-1^·s^-1^), but, in this case, the increase was due to a balanced combination of a decrease in *K*_*m*_ (3.5-fold, *K*_*m*_ = 95 ± 3 μM) and an increase in *k*_*cat*_ (5.6-fold; *k*_*cat*_ = 105.01 s^-1^). This catalytic efficiency for pyrazinamide was also 3.3-fold higher than that described for AsNic (331 mM^-1^·s^-1^) [16].

**Fig 5:**
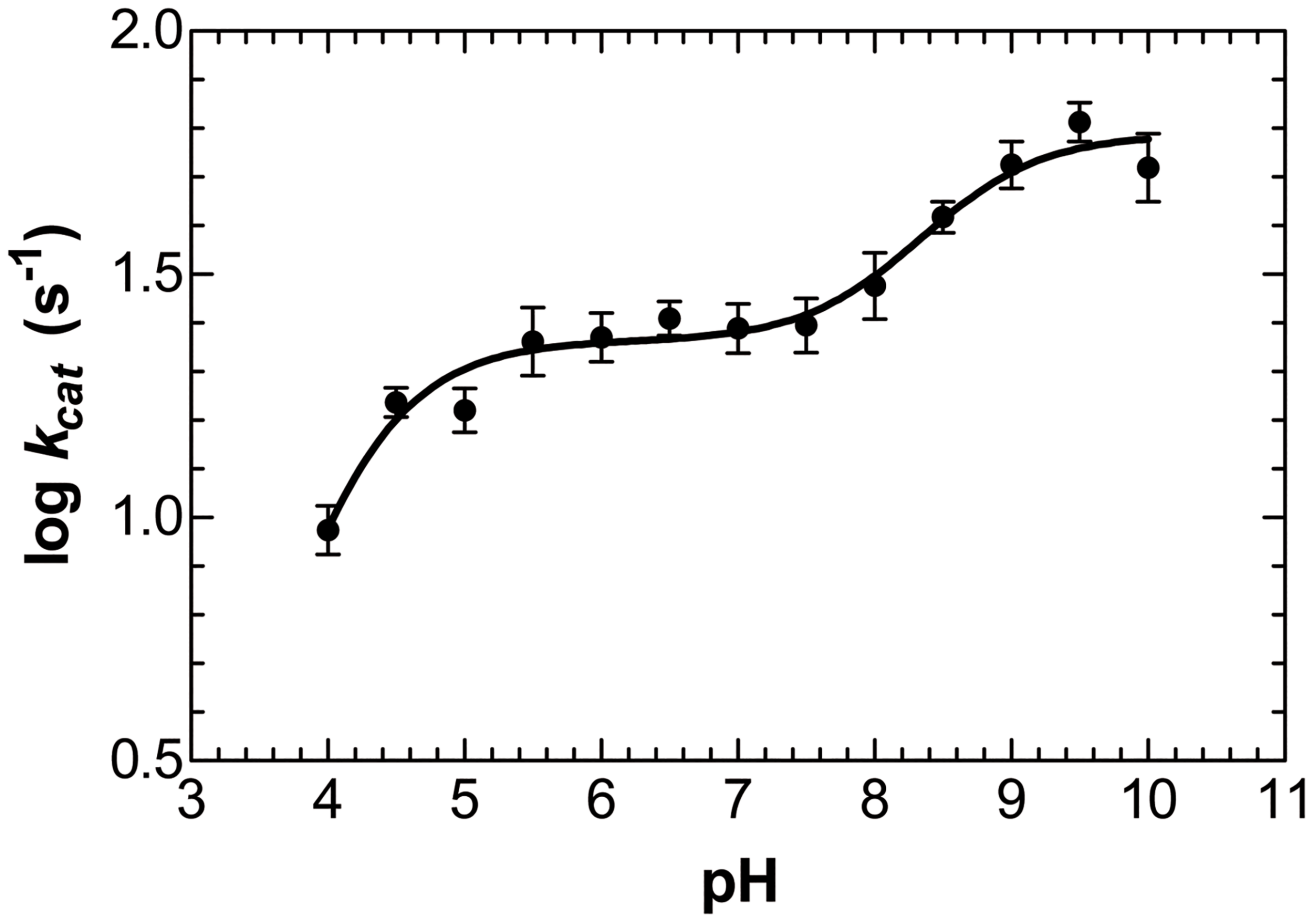
Kinetic pH rate profile of UbNic. Reactions were carried out as described in the Materials and Methods section. Data points are the average of three repeated experiments.

In order to study the particular ionization state of catalytic residues in UbNic (D25, K111 and C155), the effect of pH on the *k*_*cat*_ values was determined at a saturating nicotinamide concentration over a wide pH range (pH 4.5–10.0) (Fig 5). The pH-dependence of *k*_*cat*_ showed two catalytically relevant protonation equilibria for the enzyme substrate complex, one with an apparent p*K*_es1_ value of 4.2 ± 0.1, which must be unprotonated to be catalytically active (C155) and another p*K*_es2_ with a value of 8.6 ± 0.2 that must be protonated for activity (K111), as previously described for *Saccharomyces cerevisiae* nicotinamidase with pyrazinamide as substrate [29]. However, the difference, apart from the substrate used, is that UbNic showed a higher *k*_*cat*_ in the alkaline pH range than in the acid one. The mechanistic relevance of these results is discussed below.

### Inhibition by nicotinaldehydes

**Fig 6:**
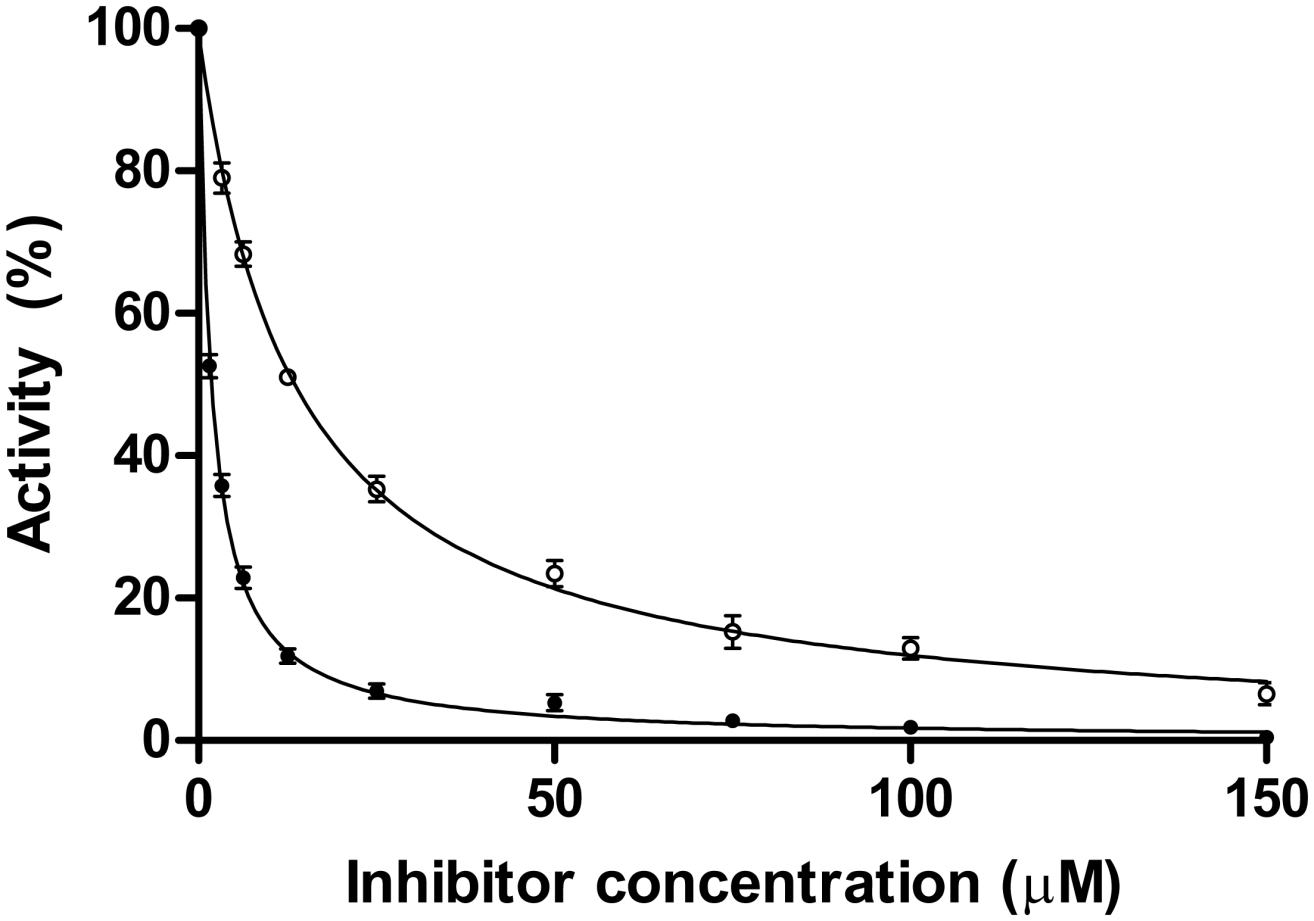
Inhibition of UbNic by nicotinaldehydes. Inhibition reactions contained 1 mM nicotinamide, 40 nM UbNic and different concentrations of nicotinaldehyde (●) or 5-bromonicotinaldehyde (○) at 37°C in 100 mM sodium phosphate pH 7.3. Morrison’s equation was used to fit data and to obtain *K*_*i*_ values, as described in Materials and Methods.

Nicotinaldehydes have been described as good competitive inhibitors for several nicotinamidases [11, 18, 29, 31, 34]. Among them, nicotinaldehyde and 5-bromo-nicotinaldehyde were the most potent and were used to show the tetrahedral thio-hemiacetal complex formed with the catalytic cysteine in SpNic during catalysis [34]. When such inhibitors were used with UbNic and nicotinamide as substrate, the *K*_*i*_ value obtained for nicotinaldehyde (180 ± 1 nM) was 8-fold lower than that found for 5-bromo-nicotinaldehyde (1470 ± 25 nM) (Fig 6), and 666- and 82-fold below the *K*_*m*_ value for nicotinamide. These results are in agreement with the results found in the thermal shift assay (Fig 4D), in which nicotinaldehyde increased *T*_*m*_ by 7°C compared with 5-bromo-nicotinaldehyde, and showed a strong binding to UbNic. This correlation between *K*_*i*_ values and the increase in *T*_*m*_ indicates that a thermal shift assay could be used as a pre-screening technique to identify new specific nicotinamidase inhibitors. When UbNic *K*_*i*_ values were compared with those described for other nicotinamidases, the *K*_*i*_ value for nicotinaldehyde was also found in the nM range, as previously reported for the nicotinamidases from *S*. *pneumoniae* (11 nM) [18], *P*. *falciparum* (34 nM) [18], *B*. *burgdorferi* (110 nM) [18], *S*. *cerevisiae* (from 1400 to 940 nM) [18, 29], *C*. *elegans* PNC1 (110 nM) and PNC2 (22 nM) [18], PolyNic (180 nM) [11] and *M*. *tuberculosis* (290 nM) [31].

### Structural analysis

**Fig 7:**
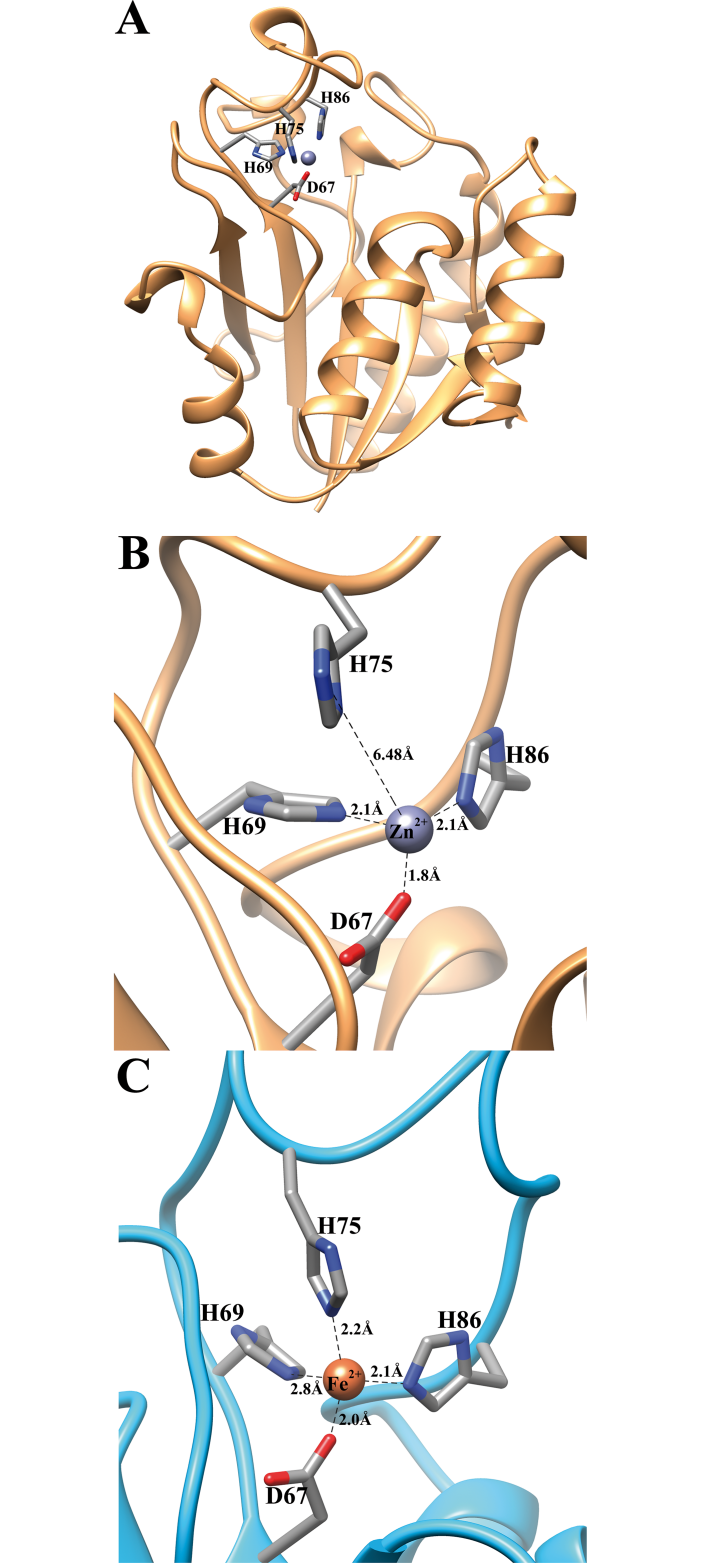
Modeled structure of UbNic. A) Ribbon diagram of the UbNic monomer and the location of the metal binding site (D67, H69, H75 and H86) obtained from SwissModel [53] using *Pyrococcus horikoshii* nicotinamidase (PDB code: 1IM5) [13] as a template. The Zn^2+^ is shown as a purple sphere. B) Detailed view of the metal binding site shown in A. C) Detailed view of the UbNic metal binding site when its sequence is modeled with SwissModel [53] using the *Mycobacterium tuberculosis* structure (PDB code: 3PL1) [33] as a template. The Fe^2+^ ion is shown as an orange sphere.

The crystal structure of the *Pyrococcus horikoshii* nicotinamidase (PDB code: 1IM5, 53% sequence identity) [13] was selected by Swiss-Model [53] to homology modeling of UbNic structure. According to this, UbNic showed the typical α/β-fold with a five-stranded β-sheet (Fig 7A) flanked by three α-helices (α_2_, α_3_, α_4_) on one side and another α-helix (α_1_) on the other side. In this UbNic model (Fig 7A), the fourth amino acid involved in metal binding (H75) was displayed in a rotamer that was far from the modeled Zn^2+^ (6.5 Å) (Fig 7B). In order to assess whether this was due to the UbNic sequence itself or the crystallographic model selected by Swiss-Model, a detailed study of the UbNic metal binding sequence was carried out using the alignment shown in Fig 1. The metal binding motif present in UbNic sequence (**D**W**H**PEQTR**H****)** was different from those described for the fourth amino acid involved in metal binding for Firmicutes (**D**A**H**XXXDXXHP**E**) [17], Archaea (**D**X**H**XXXDX**E**) [16], and the nicotinamidases from *Pyrococcus horikoshii* and *Acinetobacter baumani* (**D**W**H**PXXH) [33]. However, it is similar to that of *Mycobacterium tuberculosis* (**D**F**H**XXPXX**H**) [33], but with two main differences. In *Mycobacterium* nicotinamidases, the motif starts with a conserved **D**F**H**I sequence, whereas in UbNic and UbNic-like sequences (S3 Fig), the motif starts with a conserved **D**W**H**P sequence. The second difference is the distance between the third and fourth amino acid involved in metal binding, which is ten amino acids in UbNic compared with the thirteen found in *Mycobacterium* nicotinamidases (Fig 1). Thus, UbNic and related proteins seem to be a subgroup within the general pattern already described for *Mycobacterium* nicotinamidases.

This relationship between UbNic and *Mycobacterium* nicotinamidases metal binding [32, 33] was also found in the ion bound to the enzyme. The color of the concentrated enzyme preparation (greenish-blue) used for ICP-OES experiments (48 μM) corresponded mainly with the Fe^2+^ found (42 ± 0.06 μM), and trace amounts of Zn^2+^ (0.8 ± 0.06 μM) and Mn^2+^ (1.2 ± 0.02 μM). In fact, when UbNic was modeled with Swiss-Model, selecting the *Mycobacterium tuberculosis* structure (PDB code: 3PL1) rather than the structure of *Pyrococcus horikoshii*, the fourth amino acid involved in metal binding (H75) was correctly modeled in a rotamer that binds the bigger Fe^2+^ ion at the correct distance of 2.2 Å (Fig 7C). In addition, the above-described results also agree with the phylogenetic tree shown in Fig 1, in which UbNic is close to the *Mycobacterium* clade. Together with *Mycobacterium tuberculosis* [32, 33] and the archaeon *Acidolobus saccharovorans* [16], UbNic is the third nicotinamidase described with Fe^2+^ bound, in contrast with the nicotinamidases from *Pyrococcus horikoshii*, *Streptococcus pneumoniae*, *Streptococcus mutans*, *Saccharomyces cerevisiae Oceanobacillus iheyensis* and *Acinetobacter baumanii*, that bind Zn^2+^ ion [13, 17, 18, 20, 24, 59]. Finally, and in relation to the coordination of the metal bound to nicotinamidases, two different arrangements have been described for this class of enzymes. In the nicotinamidases from *Pyrococcus horikoshii* (PhNic) [13], *Acinetobacter baumanii* (AbNic) [20] and *Saccharomyces cerevisiae* (ScNic or ScPnc1) [24], an aspartate and two histidine residues coordinate the metal ion with the assistance of three water molecules, showing an octahedral coordination. When the substrate is bound, its pyridyl nitrogen replaces the axial water, maintaining the same octahedral geometry. In contrast, the *Mycobacterium tuberculosis* nicotinamidase (MtNic) [33] and probably UbNic, together with that of *Streptococcus pneumoniae* (SpNic) [34], coordinate their corresponding metal ion with the help of four amino acids (D67, H69, H75 and H86, UbNic numbering) and two water molecules (or one water when the ligand is bound) to give a similar octahedral arrangement. However, all of them have the presence of an equatorial water in common (just opposite to the histidine with the higher residue number) located about 5–6 Å from the carbonyl carbon of the substrate.

The function of this equatorial water has been the subject of continuous debate since the first proposed catalytic mechanism of nicotinamidases was described [13]. In that mechanism, a Zn^2+^-coordinated hydroxide from the above-mentioned equatorial water attacks the thioester intermediate formed in the deamination half-reaction, and then another incoming water replenishes the nucleophilic water with a new Zn^2+^-coordinated hydroxide with the concomitant protonation of the catalytic aspartate and the concomitant decay of the acyl intermediate to release pyrazinoic acid [13]. However, this first mechanism with two rounds of metal-ion direct water activation was seen to be inconsistent with the structural data of AbNic [20]. These authors proposed that the octahedral coordination is important to obtain the precise emplacement of the substrate and an efficient ligand exchange, the incoming water activation being only needed for the protonated catalytic aspartate to produce the hydroxide group to attack the acyl intermediate in order to yield a thiolate and nicotinic acid [20]. The above mechanism was also proposed for MtNic, in which departing ammonia is replaced by an incoming water molecule, which is then activated by the ionized carboxylate of catalytic aspartate [31]. However, French *et al*. [34] reexamined the catalytic mechanism of SpNic by means of structural, inhibition, mutagenesis and ^18^O isotope exchange studies, suggesting that (i) the catalytic lysine protonates the leaving ammonia and then facilitates the attack of the incoming water or (ii) the Zn^2+^-bound water protonates the leaving ammonia and the resulting Zn^2+^ hydroxide then activates the incoming water to attack the thioester intermediate. They also proposed that both routes for ammonia quenching in the active site may have co-evolved [18]. Later, the structural and kinetic isotope effect studies of ScNic [29] also suggested that a protonated catalytic aspartate is in the proper position to activate the deprotonation of this incoming water. In addition, QM/MM studies of SpNic showed that the active site lysine probably plays a stabilizing role in the mechanism for the thiol/thiolate intermediates [30]. However, although another QM/MM study using ScNic as a model proposed the same role for lysine and the deprotonation of incoming water by catalytic aspartate, it also concluded that the Zn^2+^-binding site rather than a single Zn^2+^ ion acts as a Lewis acid in the enzymatic reaction [60].

In line with the above questions concerning the mechanism, the results described for the *k*_*cat*_ pH-dependence of UbNic (Fig 5) with two maxima and a p*K*_es2_ of 8.6 point to the possibility that deprotonation of the catalytic lysine above pH 8.6 and/or the formation of an active site hydroxide could be a viable step in catalysis, especially taking into account that p*K*_es2_ is of a magnitude similar to that of the [Fe(H_2_O)_6_]^2-^ complex (estimated as being 8.5–9.0 and experimentally measured as 9.3–9.5) [61], and that it could also be ascribed to the deprotonation of a water ligand. A similar p*K* value (p*K*_a_ = 9.0) has been described for [Zn(H_2_O)_6_]^2-^ [61].

In addition, the protonation of the nicotinic acid oxygen in the product complex would seem very unlikely at pH 9.5–10. Thus, the results obtained may also reconcile the two main mechanisms proposed for nicotinamidases, suggesting that at non-alkaline pH values, the incoming catalytic water could be deprotonated by the catalytic aspartate, whereas at alkaline pHs, the deprotonated catalytic lysine and/or the metal ion activated hydroxide could play an active role in this crucial deprotonation step. This mechanistic flexibility may be an advantage for nicotinamidases to rapidly adapt to environmental changes, as previously described for organophosphate-degrading enzymes [47].

## Conclusions

A new thermostable metagenomic bacterial nicotinamidase from an extreme environment has been expressed, purified and characterized. Its strong expression in *E*. *coli* has allowed us to purify up to 75 mg of the protein per liter of culture with a simple procedure. The enzyme is also the most alkaliphilic nicotinamidase described to date, with high stability over a broad range of pHs and temperatures. It is also the first bacterial nicotinamidase described with a similar optimum temperature to that of the archaeon *Acidilobus saccharovorans* [16]. Furthermore, UbNic was found to contain Fe^2+^ in its metal binding site, which is coordinated by an aspartate (D67) and three histidine residues (H69, H75 and H86), in a similar way to that of *Mycobacterium tuberculosis* [33]. Of note is its catalytic efficiency toward nicotinamide, being one of the highest described in the bibliography for a non-pathogenic microorganism (nicotinamidase). All these characteristics make UbNic a versatile nicotinamidase, and suggest that substantial metabolic diversity remains to be discovered within extreme environments like those found in geothermal water stream microbial mat communities. In addition, its biphasic pH-dependence suggests a flexible catalytic mechanism for the efficient recycling of nicotinic acid into the valuable NAD^+^ redox cofactor.

## Supporting information

S1 Table Oligonucleotide sequences used for UbNic WT cloning and those used for site-directed mutagenesis. (PDF)

S2 Table Accession codes and microorganisms used in the phylogenetic tree. (PDF)

S1 Fig Pyrazinamidase activity determined by the whole-cell screening method. Fosmid clone (JFF054_F02) [38] from an uncultured bacterium [39] was assayed with the whole-cell functional screening method described for the identification of new nicotinamidases from fosmid metagenomic/polygenomic libraries as described in Materials and Methods. Fosmid clone (JFF054_F02) was compared with that of PolyNic [11]. C (-), control carried out with *E*. *coli* EPI300 cells (Epicentre) without fosmid.
(PDF)

S2 Fig Effect of substrate concentration on UbNic activity. A, B) Effect toward nicotinamide as substrate at 37°C and 60°C, respectively. C, D) Effect toward pyrazinamide as substrate at 37°C and 60°C, respectively. Reactions were carried out under the standard reaction conditions, using increasing concentrations of substrate (NAM or PZA). UbNic concentrations at 37°C and 60°C was 40 nM and 1.13 nM, respectively.
(PDF)

S3 Fig Multiple sequence alignment between UbNic and related nicotinamidases. Symbols above blocks of sequences represent the secondary structure. Springs, arrows and TT represent helices strands and strict β-turns, respectively. Strictly conserved amino acids across nicotinamidases have a red background and similar residues are marked with a rectangle. Residues involved in catalysis (▲), in *cis*-peptide bond (●) and in the metal ion binding (■) are also shown. The fourth amino acid involved in metal binding is shown as a star (★).
(PDF)
