# Supplementary material for: Biochemical characterization of a new nicotinamidase from an unclassified bacterium thriving in a geothermal water stream microbial mat community
Source: PLoS One. 2017 Jul 27;12(7):e0181561. doi: 10.1371/journal.pone.0181561 (PMC5531466; doi:10.1371/journal.pone.0181561)
Supplement: S1 Table — (PDF) [file pone.0181561.s001.pdf]

S1 Table. Oligonucleotide sequences used for UbNic WT cloning and those used for site-directed mutagenesis.

Variant	Primer sequence
UbNic $_{WT}$	5’ - TTGGGGCGCTAGCATGGGCGCAAGCGAGGCGGG - 3’
	3’ - ATCCCGCCTCGAGTCACCGGGGCGCCGCCTCGGC - 5’
H75E	5’ - ACCCGGAGCAGACGCGAGAATTCCAGGCCTACGGAGG - 3’
	3’ - TGGGCCTCGTCTGCGCTCTTAAGGACCGGATGCCTCC - 5’
H75S	5’ - ACCCGGAGCAGACGCGAAGTTTCCAGGCCTACGGAGG - 3’
	3’ - TGGGCCTCGTCTGCGCTTCAAAGGTCCGGATGCCTCC - 5’
W83F	5’ - CAGGCCTACGGAGGCCTGTTTCCTCCTCACTGCGTGCAA - 3’
	3’ - GTCCGGATGCCTCCGGACAAAGGAGGAGTGACGCACGTT - 5’
A151C	5’ - GTACGTCGGCGGGGTGTGCACTGATTACTGCGTGC - 3’
	3’ - CATGCAGCCGCCCCACACGTGACTAATGACGCACG - 5’
