# Supplementary material for: Biochemical characterization of a new nicotinamidase from an unclassified bacterium thriving in a geothermal water stream microbial mat community
Source: PLoS One. 2017 Jul 27;12(7):e0181561. doi: 10.1371/journal.pone.0181561 (PMC5531466; doi:10.1371/journal.pone.0181561)
Supplement: S2 Table — (PDF) [file pone.0181561.s002.pdf]

S2 Table. Accession codes and microorganisms used in the phylogenetic tree

Accession code	Microorganism
WP_048839456.1	*Acetobacter cibinongensis*
D9Q1R5	*Acidilobus saccharovorans*
WP_007849394.1	*Acidovorax sp.*
2WT9	*Acinetobacter baumanii*
Q6F6U3	*Acinetobacter baylyi*
SEF90419.1	*Actinomadura echinospora*
SDW29473.1	*Aidingimonas halophila*
WP_031228057.1	*Alcanivorax sp.*
WP_043764788.1	*Algiphilus aromaticivorans*
SFU13760.1	*Algoriphagus locisalis*
SEL83755.1	*Alkalibacterium putridalgicola*
A0A011QXH0	*Alkalibacterium sp.*
SEN84794.1	*Amphibacillus marinus*
WP_034274152.1	*Amycolatopsis halophila*
WP_057940983.1	*Anaerobaculum hydrogreniformans*
WP_035073133.1	*Anditalea andensis*
SFA60283.1	*Anoxybacillus pushchinoensis*
WP_010880618.1	*Aquifex aeolicus*
EEK89589.1	*Bacillus cereus*
C3AL85	*Bacillus mycoides*
WP_044738930.1	*Bacillus mycoides*
KYH41228.1	*Bathyarchaeota archaeon*
SEJ54563.1	*Bhargavaea ginsengi*
A0A0H3C0I4	*Borrelia burgdorferi*
SEN61493.1	*Brachymonas denitrificans*
Q9N426	*Caenorhabditis PNC1*
Q7YTI0	*Caenorhabditis PNC2*
WP_015233144.1	*Caldisphaera lagunensis*
XP_444840.1	*Candida glabrata*
SBO18221.1	*Carnobacterium divergens*
SDK41321.1	*Catalinimonas alkaloidigena*
WP_027103503.1	*Comamonadaceae bacterium*
AGU68151.1	*Crithidia acanthocephali*
WP_019573597.1	*Curvibacter sp.*
WP_020890380.1	*Cyclobacterium gasimii*
OGP31817.1	*Deltaproteobacteria bacterium*
WP_015723881.1	*Desulfobulbus propionicus*
SDO08672.1	*Desulfonauticus submarinus*
WP_013561684.1	*Desulfurococcus musosus*
SFM25846.1	*Ectothiorhodospira mobilis*
SET70871.1	*Enterococcus sp.*
XP_017989700.1	*Eremothecium sinecaudum*
OGS62196.1	*Euryarchaeota archaeon*
WP_020526174.1	*Flexithrix dorotheae*
WP_052464834.1	*Geoalkalibacter subterraneus*
A4IP16	*Geobacillus thermodenitrificans*
GAJ44134.1	*Geobacillus thermoglucosidasus*
WP_010942927.1	*Geobacter sulfurreducens*

KES18047.1	*Gilliamella apicola*
WP_007400460.1	*Gluconacetobacter sp.*
WP_026914784.1	*Gramella portivictoriae*
KUO42994.1	*Hadesarchaea archaeon*
WP_018129036.1	*Haladaptatus paucihalophilus*
WP_027336619.1	*Halomonas sp.*
AGU68200.1	*Herpetomonas muscarum*
WP_034819112.1	*Idiomarina sp.*
WP_012123347.1	*Ignicoccus hospitalis*
ADM28259.1	*Ignisphaera aggregans*
CCK70122.1	*Kazachstania naganishii*
WP_035848852.1	*Kitasatospora azatica*
WP_034928181.1	*Komagataeibacter rhaeticus*
WP_012309462.1	*Korarchaeum cryptofilum*
SCV04139.1	*Lachancea mirantina*
3R2J	*Leishmania infantum*
SFB04909.1	*Lentibacillus halodurans*
KPI83077.1	*Leptomonas seymouri*
SEQ07614.1	*Lewinella agarilytica*
WP_053169566.1	*Limnohabitans planktonicus*
D7WZV7	*Lysinibacillus fusiformis*
SFR43312.1	*Marinobacter gudaonensis*
WP_026609411.1	*Methylocaldum szegediense*
Q9DCC7	*Mus Musculus Isochorismatase OUTGROUP*
A0R6D1	*Mycobacterium smegmatis*
3PL1	*Mycobacterium tuberculosis*
XP_003673137.1	*Naumovozyma castellii*
OGW13671.1	*Nitrosp.irae bacterium*
WP_067900593.1	*Nocardia vaccinii*
SDJ62486.1	*Nonomuraea jiangxiensis*
Q8ESQ6	*Oceanobacillus iheyensis*
OGX43230.1	*Omnitrophica bacterium*
OGX06159.1	*Omnitrophica bacterium*
SHG09001.1	*Ornithinibacillus halophilus*
WP_028602307.1	*Ottowia thiooxydans*
SDX45676.1	*Paenibacillus sp.*
SEN76409.1	*Paenisporosarcina quisquiliarum*
WP_048152510.1	*Palaeococcus ferrophilus*
SFA40406.1	*Parageobacillus thermantarcticus*
A0A150MVW6	*Parageobacillus toebii*
WP_015899029.1	*Persephonella marina*
CCW64195.1	*Phytomonas sp.*
WP_011177472.1	*Picrophilus torridus*
A0A024VBW5	*Plasmodium falciparum*
XP_018643640	*Plasmodium gaboni*
CRG94254	*Plasmodium gallinaceum*
XP_012761276	*Plasmodium reichenowi*
PolyNic	*Polygenomic nicotinamidase*
WP_057436208.1	*Pseudomonas syringae*
SDN59408.1	*Psychrobacillus sp.*
1IM5	*Pyrococcus horikoshii*

WP_014026966.1	*Pyrolobus fumarii*
SDE12863.1	*Rhodococcus tukisamuensis*
WP_027288402.1	*Rhodovibrio salinarum*
OIZ96122.1	*Rickettsiella isopodorum*
AKQ39079.1	*Riemerella anatipestifer*
KRT71235.1	*Rokubacteria bacterium*
WP_052349027.1	*Saccharibacter sp.*
2H0R	*Saccharomyces cerevisiae*
KES10321.1	*Snodgrassella alvi*
WP_012873759.1	*Sp.haerobacter thermophilus*
WP_034088486.1	*Streptacidiphilus albus*
3S2S	*Streptococcus mutans*
3O90	*Streptococcus pneumoniae*
WP_037907090.1	*Streptomyces yeochonensis*
KUK40212.1	*Synergistales bacterium*
WP_048850081.1	*Tanticharoenia sakaeratensis*
L0EFU6	*Thermobacillus composti*
WP_068758442.1	*Thermobifida cellulosilytica*
WP_068576905.1	*Thermococcus chitonophagus*
WP_028847923.1	*Thermocrisp.um agreste*
WP_012643066.1	*Thermomicrobium roseum*
WP_010900881.1	*Thermoplasma acidophilum*
XP_003682297.1	*Torulaspora delbrueckii*
KHN79436.1	*Toxocara canis*
H5SPS2	*UbNic*
WP_068630822.1	*Variovorax sp.*
SDH49011.1	*Vibrio xiamenensis*
SHH13069.1	*Virgibacillus chiguensis*
CDH13574.1	*Zygosaccharomyces bailii*
