# Supplementary figures and images for: Biochemical characterization of a new nicotinamidase from an unclassified bacterium thriving in a geothermal water stream microbial mat community
Source: PLoS One. 2017 Jul 27;12(7):e0181561. doi: 10.1371/journal.pone.0181561 (PMC5531466; doi:10.1371/journal.pone.0181561)

## Autoinduction solution (-)

**C (-)**

**PolyNic**

**UbNic**

## Autoinduction solution (+)

**C (-)**

**PolyNic**

**UbNic**

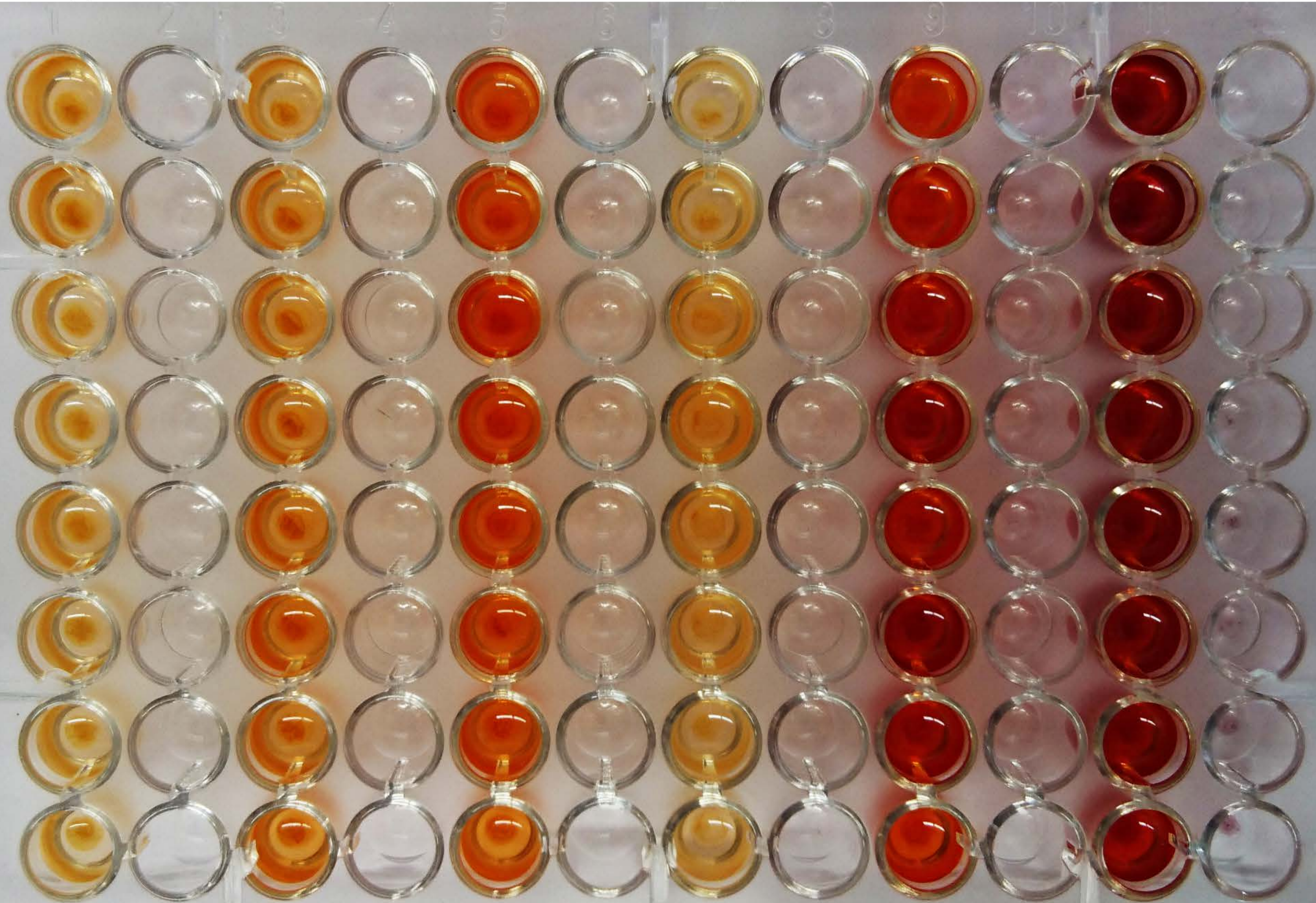

Supplement: S1 Fig — Fosmid clone (JFF054_F02) [38] from an uncultured bacterium [39] was assayed with the whole-cell functional screening method described for the identification of new nicotinamidases from fosmid metagenomic/polygenomic libraries as described in Materials and Methods. Fosmid clone (JFF054_F02) was compared with that of PolyNic [11]. C (-), control carried out with E. coli EPI300 cells (Epicentre) without fosmid. (PDF) [file pone.0181561.s003.pdf]

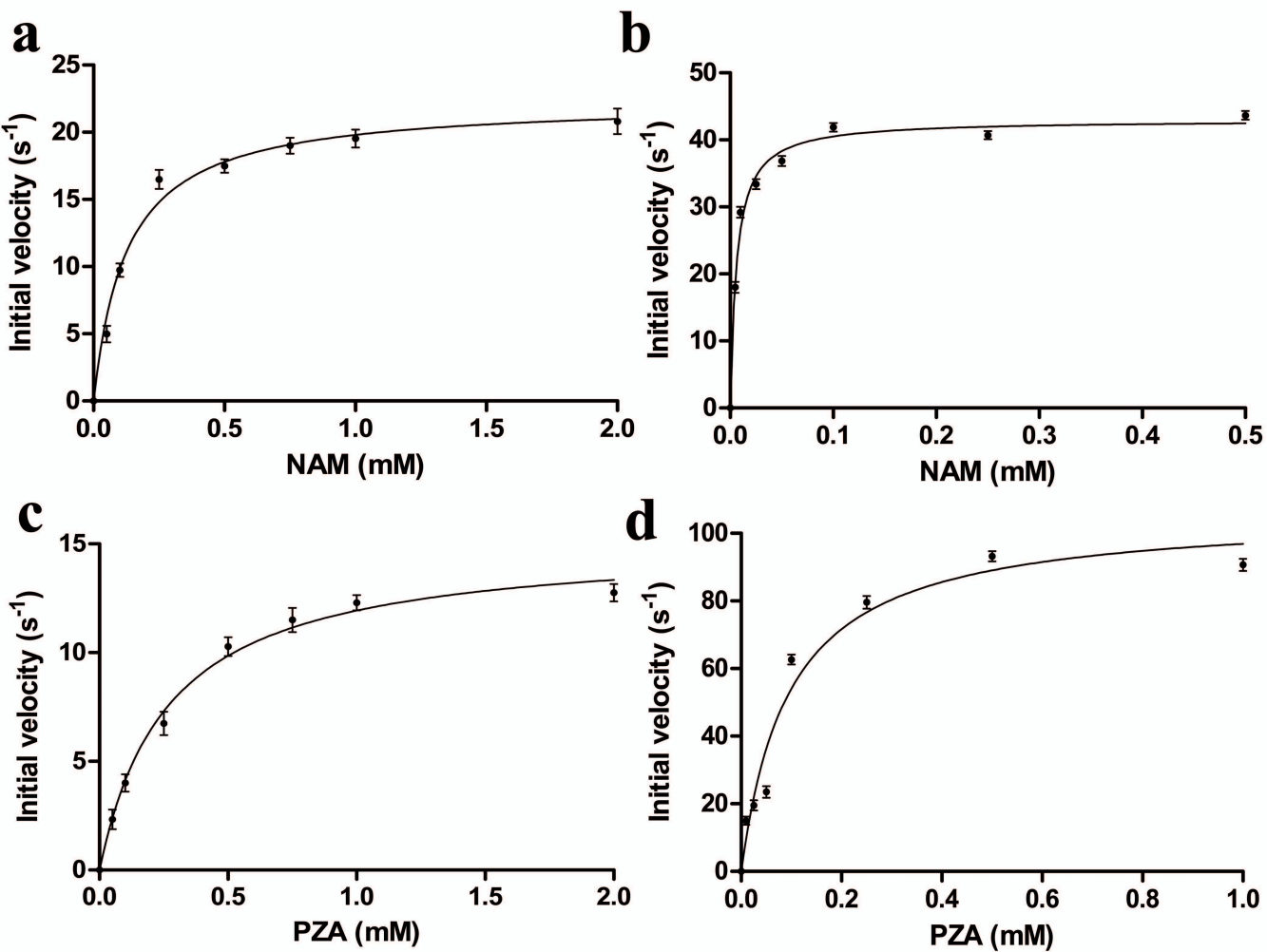

Supplement: S2 Fig — A, B) Effect toward nicotinamide as substrate at 37°C and 60°C, respectively. C, D) Effect toward pyrazinamide as substrate at 37°C and 60°C, respectively. Reactions were carried out under the standard reaction conditions, using increasing concentrations of substrate (NAM or PZA). UbNic concentrations at 37°C and 60°C was 40 nM and 1.13 nM, respectively. (PDF) [file pone.0181561.s004.pdf]

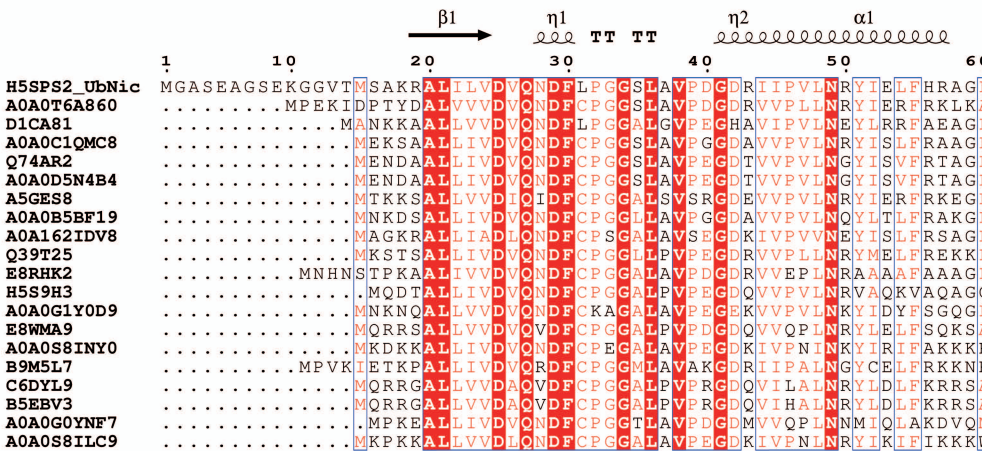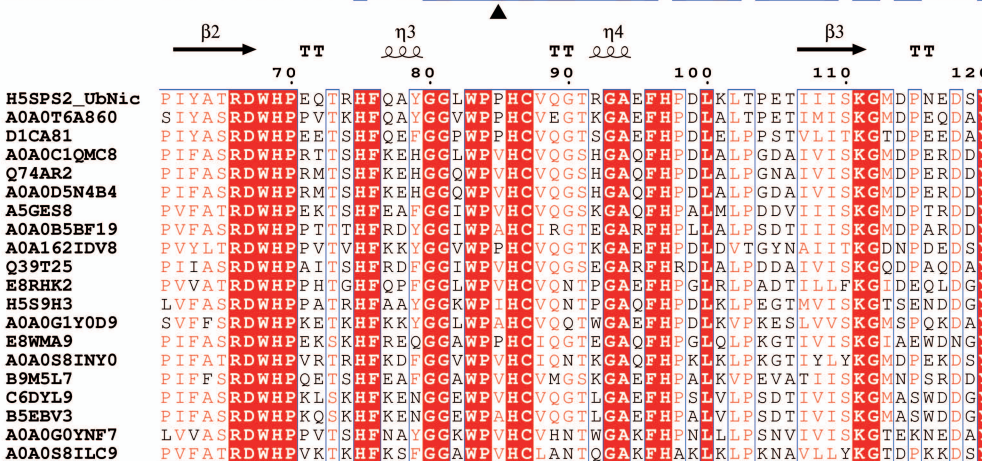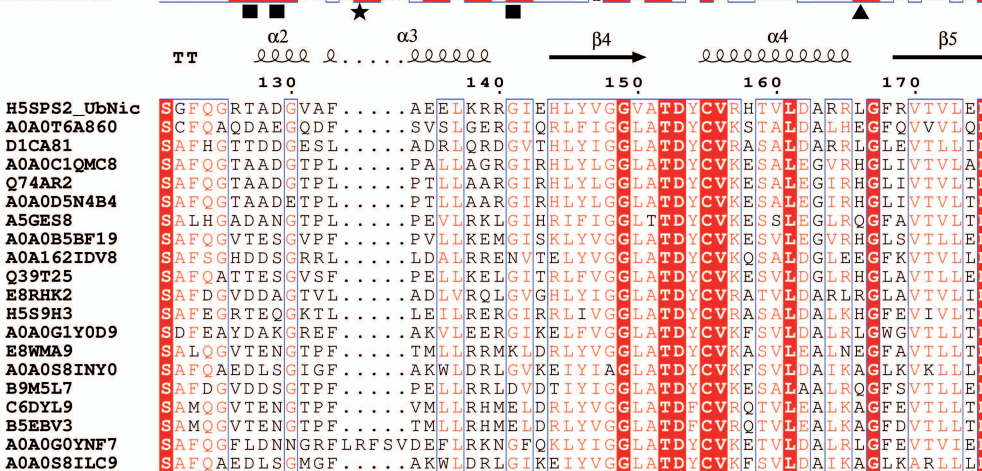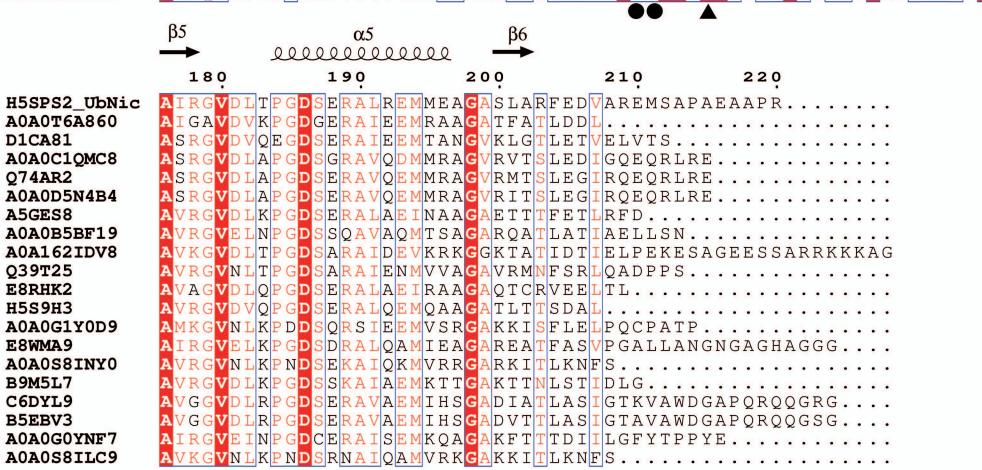

Supplement: S3 Fig — Symbols above blocks of sequences represent the secondary structure. Springs, arrows and TT represent helices strands and strict β-turns, respectively. Strictly conserved amino acids across nicotinamidases have a red background and similar residues are marked with a rectangle. Residues involved in catalysis (▲), in cis-peptide bond (●) and in the metal ion binding (■) are also shown. The fourth amino acid involved in metal binding is shown as a star (★). (PDF) [file pone.0181561.s005.pdf]
